# Defect-Engineered Elastic CNC/Chitosan-Based Carbon Aerogel with Wideband Microwave Absorption

**DOI:** 10.3390/nano15161233

**Published:** 2025-08-13

**Authors:** Weikai Zhan, Yijie Hu, Liangjun Li, Yonggang Jiang, Junzong Feng, Jian Feng

**Affiliations:** Science and Technology on Advanced Ceramic Fibers and Composites Laboratory, College of Aerospace Science and Engineering, National University of Defense Technology, Changsha 410073, China; zhanweikai19@nudt.edu.cn (W.Z.); llj19840604@163.com (L.L.); jygemail@nudt.edu.cn (Y.J.); junzongfeng@nudt.edu.cn (J.F.); fengj@nudt.edu.cn (J.F.)

**Keywords:** carbon aerogel, cellulose nanocrystal, compressive elasticity, microwave absorption, defect engineering

## Abstract

The burgeoning electromagnetic pollution from 5G/6G technologies demands lightweight, broadband, and mechanically robust electromagnetic microwave absorbers (EMWAs). Conventional carbon aerogels suffer from structural fragility and inadequate electromagnetic dissipation. Herein, we propose a defect-engineering strategy through precise optimization of the chitosan (CS)/cellulose nanocrystal (CNC) ratio to fabricate elastic boron nitride nanosheet (BNNS)-embedded carbon aerogels. By fixing BNNS content for optimal impedance matching and modulating the CS/CNC ratio of the aerogel, we achieve synergistic control over hierarchical microstructure, defect topology, and electromagnetic response. The aerogel exhibits a wide effective absorption bandwidth (EAB) of 8.3 GHz at a thickness of 3.6 mm and an excellent reflection loss of −52.79 dB (>99.999% attenuation), surpassing most biomass-derived EMWAs. The performance stems from CNC-derived topological defects enabling novel polarization pathways and BNNS-triggered interfacial polarization, while optimal graphitization (I_D_/I_G_ = 1.08) balances conductive loss. Simultaneously, the optimal CS/CNC ratio facilitates the formation of a stable and flexible framework. The long-range ordered micro-arch lamellar structure endows the aerogel with promising elasticity, which retains 82% height after 1000 cyclic compression at 50% strain. This work paves the way for biomass-derived carbon aerogels as next-generation wearable and conformal EMWAs with broadband absorption.

## 1. Introduction

The proliferation of 5G/6G high-frequency communication technologies has intensified electromagnetic radiation pollution from electronic devices, resulting in significant risks to human health and information security. Addressing this challenge necessitates developing electromagnetic microwave absorbers (EMWAs) with lightweight, broadband, and strong absorption characteristics. Conventional carbon aerogels usually reveal structural fragility [1], severely limiting their practical performance. To meet electromagnetic protection demands for wearable devices, flexible carbon aerogels with superior wave absorption capabilities are imperative. Current research primarily focuses on incorporating exogenous functional fillers [2], including boron nitride nanosheets (BNNS), reduced graphene oxide (rGO), and MXene into biomass-derived carbon matrices. However, optimizing the intrinsic structural characteristics of biomass matrix components remains underexplored [3].

Recent advances in polymer-based EMWA composites underscore the pivotal role of conductive fillers in tailoring electromagnetic properties. Jang et al. [4] provided a comprehensive analysis of how diverse carbon nanomaterials (e.g., carbon nanotubes, graphene) influence electromagnetic wave (EMW) absorption performance within polymer matrices, highlighting the critical interplay between filler type, morphology, and loading in determining impedance matching and loss mechanisms. Similarly, bilayer core–shell structures (e.g., NiFe_2_O_4_@BiFeO_3_@polypyrrole) have demonstrated enhanced low-frequency absorption (2–8 GHz) by leveraging interface-induced dual-pinning effects, achieving a reflection loss (RL) of −65.3 dB at 4.43 mm [5]. Self-healing polyurethane/MOF composites with boron ester bonds exhibit RL values of −47.1 dB at 12.8 GHz while recovering structural integrity after damage [6]. However, a significant challenge persists: the incorporation of conductive fillers, while often improving attenuation, can readily disrupt impedance matching. This mismatch shifts the dominant interaction mechanism from desirable wave absorption towards detrimental reflection, effectively transforming the material into an electromagnetic shield rather than an absorber.

Cellulose nanocrystal (CNC), as a natural one-dimensional nanomaterial, possesses high crystallinity and a rigid chain structure. During carbonization, CNCs anchor polymer networks and mitigate excessive chitosan shrinkage [7]. Under bidirectional freezing, these components facilitate the formation of highly ordered wavelike micro-arch lamellar structures [8]. Zhuo et al. [9] integrated CNC and konjac glucomannan to construct lightweight and highly elastic carbon aerogels. Zhai et al. [10] constructed a three-dimensional framework by blending waterborne polyurethane with CNC, endowing the composite aerogel with flexible mechanical properties and sensing performance. Furthermore, CNC-derived carbon skeletons contain topological defects that provide novel polarization loss pathways [11], overcoming interfacial polarization limitations. Thus, CNC serves dual roles: structural stabilization and enhanced dielectric dissipation.

In this study, BNNS/chitosan (CS)/CNC carbon aerogels (BCC aerogels) were fabricated through precise CS/CNC mass ratio control. BNNS incorporation ensured impedance matching, and the role of CNC in modulating architecture, defect density, and microwave absorption was systematically investigated. Attributed to its ordered micro-arch lamellae and high defect density, the aerogel achieves an ultra-wide effective absorption bandwidth (EAB, RL < −10 dB) of 8.3 GHz at 3.6 mm thickness at CS:CNC = 4:2. The structure also leads to mechanical elasticity, and the aerogel shows 82% height retention and 70% stress retention after 1000 compression cycles at 50% strain. This nanomaterial-directed microstructural design concurrently boosts mechanical robustness and optimizes electromagnetic absorption of the biomass-derived aerogel, presenting an elegant paradigm for lightweight, intelligent wearable absorbers.

## 2. Materials and Methods

### 2.1. Materials

Chitosan (CS, deacetylation degree > 95%, viscosity: 100–200 mPa·s) was purchased from Shanghai Macklin Biochemical Co., Ltd. (Shanghai, China). Cellulose nanocrystals (CNC, average diameter 5–20 nm, length: ~200 nm) were obtained from ScienceK Technology Co., Ltd. (Hsinchu County, Taiwan, China). Trisodium citrate dihydrate (C6H5Na3O7, AR) and glacial acetic acid (CH3COOH, AR) were obtained from Guoyao Chemical Reagent Co., Ltd. (Guangzhou, China). Hexagonal Boron Nitride Nanosheets (BNNS, average diameter 1–5 μm, thickness: <5 nm) were supplied by Jiangsu Xianfeng nanomaterials technology Co., Ltd. (Nanjing, China). Ultrapure water was used during the experiments. All chemicals were used as received without any further purification.

### 2.2. Fabrication of BCC Aerogels

A CS solution was prepared by dissolving chitosan powder in deionized water containing 4.0 wt% glacial acetic acid and sodium citrate (to facilitate dissolution). Concurrently, a CNC suspension was obtained by dispersing CNC powder in deionized water. Separately, BNNS were dispersed in ultrapure water at a fixed concentration and sonicated for 20 min using an LC-AUD ultrasonic processor (1000 W) (Shanghai Lichen Instrument Technology Co., Ltd., Shanghai, China) to achieve a homogeneous dispersion.

**Figure 1:**
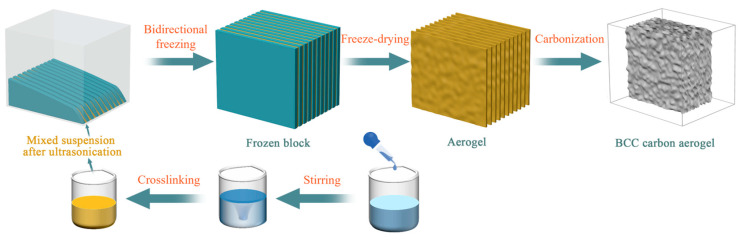
Preparation process of BCC aerogels: suspension formulation, bidirectional freeze-drying, and carbonization.

The CS solution and CNC suspension were combined in varying mass ratios and homogenized with a fixed quantity of BNNS dispersion (0.3 wt%) under magnetic stirring (800 rpm, 10 min), followed by sonication for 15 min to ensure homogeneity. The compositions of the resulting composite suspensions are summarized in Table 1 with corresponding sample labels. The fabrication of BCC aerogels is depicted in Figure 1. The resulting suspensions were transferred into molds and directionally frozen using a liquid nitrogen-assisted bidirectional freezing technique. Following complete solidification, the samples were freeze-dried at −60 °C and 1 Pa for 72 h in an LGJ-18 freeze dryer. Finally, thermal carbonization was performed at 700 °C for 1 h in a tube furnace under an argon atmosphere to produce the carbon composite aerogels.

### 2.3. Characterizations

Microstructural analysis was conducted by using field-emission scanning electron microscopy (FESEM, Regulus 8240, Hitachi High-Tech Corporation, Tokyo, Japan) at 3 kV and 10 μA. Structural characteristics and chemical bonding of carbon constituents were probed by using Raman spectroscopy (Horiba LabRAM HR Evolution, HORIBA Scientific, Kyoto, Japan, λ = 532 nm, range: 50–4000 cm^−1^). Elemental composition and chemical states were assessed by X-ray photoelectron spectroscopy (XPS, Thermo Scientific Nexsa, Thermo Fisher Scientific, Waltham, MA, USA).

Uniaxial compression tests utilized a universal testing machine (DR-5010AS, Shimadzu Corporation, Kyoto, Japan) equipped with a 10 N load cell. Samples were compressed perpendicular to the micro-arch lamellar structure at a crosshead speed of 10 mm·min^−1^, with stress/strain profiles recorded at maximum compressive strains of 10%, 30%, 50%, and 75%. Cyclic compression employed a speed of 15 mm·min^−1^. For electromagnetic characterization, aerogels were vacuum-infiltrated with paraffin wax and machined into toroidal specimens (outer diameter: 7.00 mm, inner diameter: 3.04 mm). Complex permittivity (εr) and permeability (μr) spectra were measured over 2~18 GHz by using a vector network analyzer (Rohde & Schwarz ZNA43, Rohde & Schwarz GmbH & Co. KG, Munich, Germany). The reflection loss (RL) values are used to represent the EMW absorption intensity of materials, which can be obtained by transmission line theory and calculated by the following formula:

(1) $$Z_{in}=Z_{0}\sqrt{\left|\mu_{r}/\varepsilon_{r}\right|}tanh\left[j\left(\frac{2\pi fd}{c}\right)\sqrt{\mu_{r}\varepsilon_{r}}\right],$$

(2) $$RL=20{log}_{10}\left|\frac{z_{in}-z_{0}}{z_{in}+z_{0}}\right|,$$

where *Z_in_* represents the normalized input impedance of the absorber, *Z*_0_ is the impedance of free space, *μ_r_* is the relative complex permeability (*μ_r_* = *μ*′ − *jμ*″), *ε_r_* is the complex permittivity (*ε_r_* = *ε*′ − *jε*″), *d* is the thickness of absorber, and *c* and *f* are velocity and microwave frequency, respectively.

The attenuation constant (α) can be calculated by the following formula:

(3) $$\alpha =\frac{\sqrt{2}\pi f}{c}\times \sqrt{\left(\mu^{\prime \prime }\varepsilon^{\prime \prime }-\mu^{\prime }\varepsilon^{\prime }\right)+\sqrt{{\left(\mu^{\prime \prime }\varepsilon^{\prime \prime }-\mu^{\prime }\varepsilon^{\prime }\right)}^{2}+{\left(\mu^{\prime }\varepsilon^{\prime \prime }+\mu^{\prime }\varepsilon^{\prime \prime }\right)}^{2}}},$$

*μ_r_* ≈ 1 was assumed due to the non-magnetic nature of all samples.

## 3. Results and Discussion

### 3.1. Microstructural Composition and Morphology of BCC Aerogels

**Figure 2:**
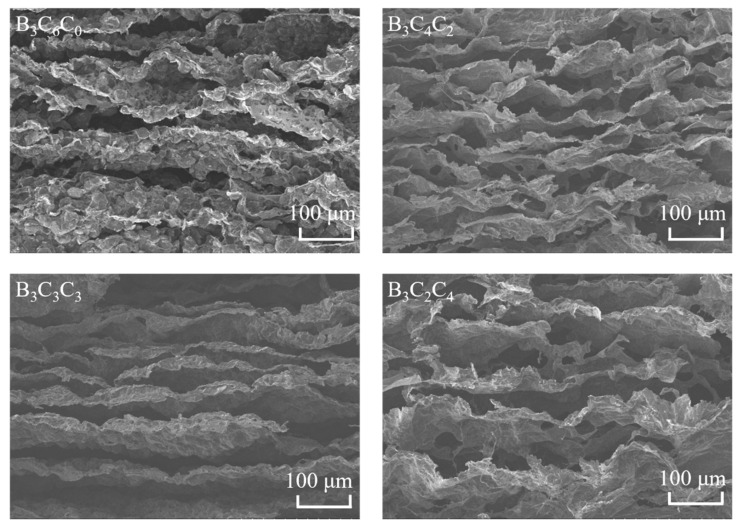
SEM images of the BCC aerogel.

SEM analysis (Figure 2) reveals distinct morphological evolution in BCC aerogels with increasing CNC content. The B_3_C_6_C_0_ aerogel (without CNC) exhibits an indistinct network skeleton, discontinuous lamellae, and pronounced surface granularity, attributed to its insufficient crosslinking due to the absence of CNC support. When the CS:CNC ratio increases to 4:2 (B_3_C_4_C_2_), a clear long-range ordered micro-arch structure is formed. Further increasing CNC content (B_3_C_3_C_3_, equal CS/CNC/BNNS ratios) yields thickened lamellae with reduced undulation, resulting from the larger CS-CNC skeletal framework and BNNS-induced lamellar coarsening. However, at a CS:CNC ratio of 2:4 (B_3_C_2_C_4_), excessive CNC content couples with insufficient CS crosslinking, which generates numerous micropores and compromised structural regularity, ultimately diminishing the lamellar strength and mechanical robustness of the aerogel.

**Figure 3:**
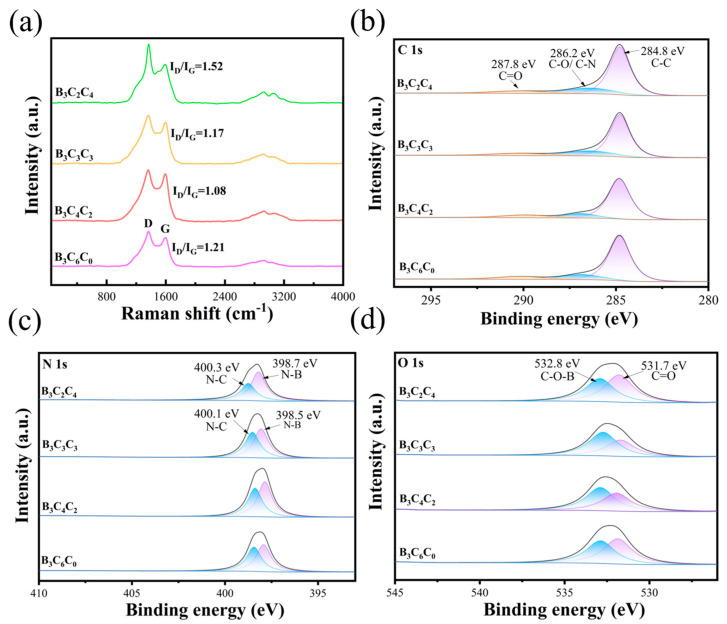
(**a**) Raman spectra and (**b**–**d**) C 1s, N 1s, and O 1s XPS spectra of the BCC aerogels, respectively.

Raman spectroscopy was employed to investigate the defect states and doping characteristics of the materials. As shown in Figure 3a, all samples exhibit two characteristic peaks approximately at 1360 cm^−1^ and 1570 cm^−1^, corresponding to the D-band and G-band of carbon materials, respectively. The D-band originates from lattice defects in amorphous carbon structures, while the G-band arises from the in-plane stretching vibrations of sp^2^-hybridized graphitic carbon. The intensity ratio of D-band to G-band (I_D_/I_G_) serves as a critical indicator for evaluating defect density and graphitization degree in carbon-based materials [12]. It reveals that the CS:CNC ratio critically modulates the defect density (I_D_/I_G_ ratio) of the resultant carbon aerogels. Specifically, the introduction of CNC generally increases the defect density (evidenced by an elevated I_D_/I_G_ ratio) within a certain range [13], and a CS:CNC ratio = 4:2 yields B_3_C_4_C_2_ with minimal I_D_/I_G_ (1.08). This minimal ratio indicates the highest graphitization degree and the lowest defect density achievable. This trend suggests that insufficient CNC content leads to inadequate structural support within the BCC framework, while excessive CNC with insufficient CS induces weak crosslinking and structural distortion within the aerogel skeleton [14]. Generally, a lower I_D_/I_G_ ratio correlates with a higher electrical conductivity and conductive loss. To prevent excessive conductivity from compromising impedance matching while simultaneously enhancing polarization loss, BNNS were incorporated into the aerogel [15]. The ordered lamellar carbon skeleton of B_3_C_4_C_2_ effectively maintains conductive loss, and the formation of efficient heterogeneous interfaces between the carbon skeleton and BNNS-promoted interfacial polarization [16]. Consequently, enhanced polarization effects are achieved without requiring high skeleton defect density, establishing an optimal balance between impedance matching and diverse loss mechanisms.

XPS analysis confirms that CNC content variations modulate chemical bonding and interfacial interactions within the aerogels [17]. The C 1s high-resolution spectrum (Figure 3b) of the B_3_C_4_C_2_ sample exhibits a notably higher intensity of the graphitic carbon peak (284.8 eV) compared to samples with other ratios [18]. Concurrently, the proportions of C-O/C-N bonds (286.2 eV) and carbonyl (C=O) groups (287.8 eV) remained relatively balanced. This indicates that the optimal CNC incorporation enhances the continuity of the carbon skeleton and modulates defect density via crosslinking between CNC hydroxyl groups and chitosan amino groups [19]. This preserves the conductive network while providing polarization sites. As the CNC ratio increases, the C 1s spectra of B_3_C_3_C_3_ and B_3_C_2_C_4_ show a progressive rise in C-O peak intensity [20]. This suggests that excess CNC disrupts the carbon layer ordering, introducing excessive insulating functional groups due to its high oxygen content, thereby impairing electron transport pathways [21]. The N 1s spectra (Figure 3c) further support this mechanism: B_3_C_4_C_2_ displays two distinct peaks at 398.5 eV (B-N) and 400.1 eV (graphitic N), with the B-N component being dominant [22]. This confirms the effective doping of chitosan-derived nitrogen into the carbon lattice. In contrast, the N 1s peak of the high-CNC-content sample (B_3_C_2_C_4_) shifts to a higher binding energy (401.3 eV), attributed to oxidation-induced electron loss of nitrogen species [23]. This shift indicates hindered pyrolysis of chitosan and reduced nitrogen doping efficiency under excess CNC conditions. O 1s spectra (Figure 3d) reveal that the C-O-B bridging bond peak (532.8 eV) intensity is higher in B_3_C_4_C_2_ than in high-CNC-content samples, suggesting that moderate CNC content strengthens polarization loss by forming interfacial bridging bonds between hydroxyl groups and boron atoms at BN edges [24]. Conversely, excess CNC leads to condensation of free hydroxyl groups, weakening the chemical anchoring between BN and carbon layers, and consequently diminishing interfacial polarization capability.

### 3.2. Elastic Mechanical Properties of BCC Aerogels

**Figure 4:**
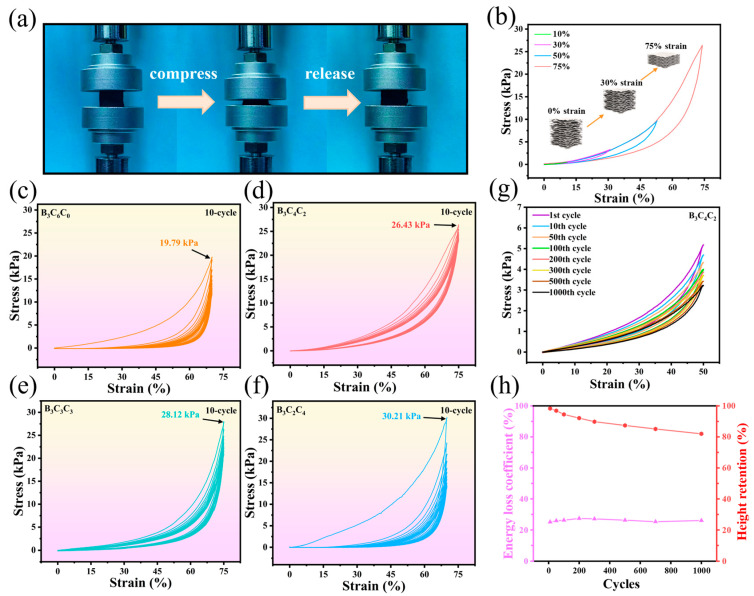
(**a**) Optical photograph of a BCC aerogel during compression; (**b**) stress/strain cyclic curves under different strains; (**c**–**f**) stress/strain curves of different BCC samples during 10 cyclic compression at 75% strain; (**g**) fatigue resistance test of B_3_C_4_C_2_; (**h**) energy dissipation coefficient and height retention of B_3_C_4_C_2_ during fatigue resistance test.

Ideal compressibility and mechanical elasticity are prerequisites for maintaining consistent EMW absorption performance after repeated compression. Uniaxial compression tests were employed to evaluate the longitudinal mechanical properties of the BCC aerogels. Figure 4a displays optical images of the B_3_C_4_C_2_ aerogel during compression. Remarkably, B_3_C_4_C_2_ withstands large deformations without fracture and recovers instantaneously to its initial state upon load release. Stress/strain curves (Figure 4b) were recorded during consecutive loading/unloading cycles at strains of 0~10%, 30%, 50%, and 75%. The curves exhibit high reproducibility across cycles, with a stress of approximately 26 kPa at 75% strain. This elasticity originates from the long-range micro-arch lamellar structure [25], where lamellae extend under compression to dissipate stress. This structure arises from the differential shrinkage between CS and CNC during high-temperature carbonization [26]. Under compression, the lamellae extend to provide effective stress buffering. Concurrently, CNC and BNNS enhance the lamellar strength and toughness, enabling greater stress accommodation and facilitating rapid elastic recovery. The stress/strain curves in Figure 4c–f show that the B_3_C_4_C_2_ demonstrates a maximum stress of 26.43 kPa at 75% strain and high stress retention (~90% after 10 cyclic compression). In contrast, B_3_C_6_C_0_ exhibits lower stress at 75% strain (19.79 kPa) and significant degradation after ten cycles. This poor elasticity stems from its matrix structure, formed solely by CS crosslinking, which results in flatter, less continuous lamellae. Increasing CNC content (B_3_C_3_C_3_ and B_3_C_2_C_4_) progressively elevates the maximum stress of BCC aerogel due to the high modulus of CNC, which reinforces the lamellae. However, their stress retention markedly decreases [27]. At high strains, the stronger CNC-rich lamellae become more susceptible to damage as the reduced CS content weakens the crosslinking efficacy, leading to diminished elasticity.

To elucidate the elastic mechanism, the exemplary B_3_C_4_C_2_ sample (Figure 4d) was analyzed. Its loading/unloading curve exhibits three distinct regimes [28]: (I) a near-linear region below ~15% strain, attributed to the reduction in inter-lamellar spacing with minimal contact between micro-arches; (II) a relatively flat plateau between 15 and 40% strain, where increased lamellar contact and post-buckling deformation of the wavelike micro-arch structure occur with minimal stress increase; (III) a densification regime above 40% strain, characterized by a rapid modulus increase due to the depletion of structural buffering as lamellae compact. The compression fatigue resistance of B_3_C_4_C_2_ was assessed via 1000 cycles at 50% strain and the sample maintains elastic recovery (Figure 4g). Figure 4h reveals that the energy dissipation coefficient (η) of the aerogel remains stable at ~20%, and the aerogel retains over 80% of its original height, indicating a mechanical durability. The high compressibility of B_3_C_4_C_2_ primarily stems from its stable, continuous micro-arch lamellar structure, enabled by the optimal CNC content, which effectively absorbs stress through lamellar extension.

### 3.3. Electromagnetic Wave Absorption Properties of BCC Aerogels

**Figure 5:**
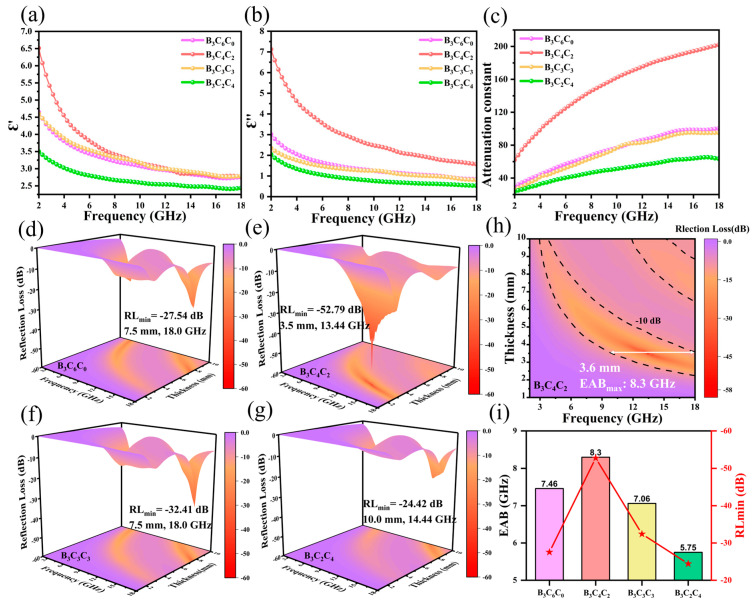
(**a**) Real part of permittivity of the BCC aerogels; (**b**) imaginary part of permittivity of the BCC aerogels; (**c**) attenuation constant comparison of the BCC aerogels; (**d**–**g**) 3D RL plots of the BCC aerogels; (**h**) 2D RL contour map of B_3_C_4_C_2_; (**i**) RL and EAB performance comparison of the four samples.

To investigate the EMW absorption properties of BCC aerogels, their complex permittivity was measured across the 2~18 GHz frequency range. The electromagnetic wave absorption performance of a material is governed by its relative complex permittivity (*ε_r_* = *ε*′ − *jε*″) and complex permeability (*μ_r_* = *μ*′ − *jμ*″). Here, the real parts (*ε*′, *μ*′) signify the material’s ability to store incident electromagnetic energy, while the imaginary parts (*ε*″, *μ*″) represent its energy dissipation capability [29]. As no magnetic components were introduced into the BCC matrix, the real and imaginary parts of the BCCs’ complex permeability [30] remained approximately 1 and 0, respectively. Consequently, this study focuses solely on the relative complex permittivity and dielectric loss of the BCCs. Figure 5a,b depict the frequency-dependent real (*ε*′) and imaginary (*ε*″) permittivity components for different materials. All curves exhibit a declining trend with increasing frequency, consistent with typical frequency dispersion behavior [31] arising from the delayed polarization response of electric dipoles under an alternating electric field. Compared to sample B_3_C_6_C_0_, sample B_3_C_4_C_2_ (with a higher CNC ratio) displays significantly enhanced *ε*′ and *ε*″ values. This enhancement stems from CNC facilitating the construction of a more ordered aerogel skeleton by CS, enriching conductive pathways and thereby strengthening conductive loss—the dominant loss mechanism [32]. Concurrently, defects induced by the CNC itself contribute to interfacial polarization, further elevating both *ε*′ and *ε*″ [33]. However, a further increase in the CNC ratio leads to a marked decrease in both *ε*′ and *ε*″, with values converging towards and subsequently falling below those of B_3_C_4_C_0_. This reduction is attributed to excessive CNC causing over-crosslinking and structural distortion within the aerogel skeleton [34]. The concomitant decrease in CS proportion also diminishes the continuity of the conductive sheets, impairing the conditions necessary for effective conductive loss. The attenuation constant (α) quantifies a material’s ability to attenuate electromagnetic energy, where higher α values correspond to stronger attenuation capabilities [35]. As shown in Figure 5c, the trend in α with increasing CNC ratio mirrors that of *ε*′ and *ε*″. Sample B_3_C_4_C_2_ (CS:CNC = 4:2) exhibits the maximum α value, indicating the strongest attenuation ability. This finding aligns with the preceding analysis of the material’s loss mechanisms.

EMW absorption properties were evaluated via transmission line theory [36]. Typically, a reflection loss (RL) ≤ −10 dB (indicating over 90% microwave attenuation) defines effective microwave absorption [37], and the corresponding frequency range is termed the effective absorption bandwidth (EAB). The RL values for all aerogel samples across different frequencies and thicknesses are presented as 3D plots and 2D contour maps in Figure 5d–g and Figure 5h, respectively.

B_3_C_6_C_0_ achieves a minimum reflection loss (RL_min_) of −27.54 dB at 18.0 GHz with a thickness of 7.5 mm. Its maximum EAB (EAB_max_) reaches 7.46 GHz at a matching thickness of 8.8 mm. The B_3_C_4_C_2_ sample (CS:CNC = 4:2) exhibits optimal EMW absorption performance, with a significantly lower RL_min_ of −52.79 dB (corresponding to >99.999% microwave attenuation) at 13.44 GHz and thickness of 3.5 mm. Concurrently, its EAB_max_ broadens substantially to 8.3 GHz at a matching thickness of 3.6 mm. This enhanced performance aligns with XPS and Raman analysis, confirming that the optimal CNC ratio enables the formation of an ordered micro-arch lamellar structure, achieving a balance between multiple loss mechanisms and impedance matching. B_3_C_3_C_3_ shows an RL_min_ of −32.41 dB at 18.0 GHz (7.5 mm thickness), with an EAB_max_ of 7.06 GHz at 8.7 mm. Further increasing the CNC content leads to a deteriorated EMW absorption performance. B_3_C_2_C_4_ exhibits an RL_min_ of −24.42 dB at 14.44 GHz (10.0 mm thickness), and its EAB_max_ narrows to 5.75 GHz at 9.5 mm. The comparison of RL and EAB performance for the four samples (Figure 5i) clearly illustrates the trend in EMW absorption performance with increasing CNC:CS ratio, and the sample B_3_C_4_C_2_ demonstrates the best RL_min_ and EAB_max_. Table 2 summarizes the key EMW absorption performance metrics of the prepared samples in this work and compares them with representative state-of-the-art biomass-derived carbon absorbers reported recently. The B_3_C_4_C_2_ demonstrates outstanding EMW absorption performance, achieving a strong RL_min_ of −52.79 dB and, crucially, a broad EAB_max_ of 8.3 GHz. While the optimal matching thickness (3.6 mm) for B_3_C_4_C_2_ falls within a typical range for high-performance absorbers, its EAB_max_ significantly surpasses that of most counterparts, which typically exhibit narrower bandwidths (often < 6 GHz) even at comparable or thinner thicknesses. This combination of deep absorption strength and wide bandwidth establishes the superior comprehensive performance of B_3_C_4_C_2_, outperforming most current biomass-based carbon absorbers.

**Figure 6:**
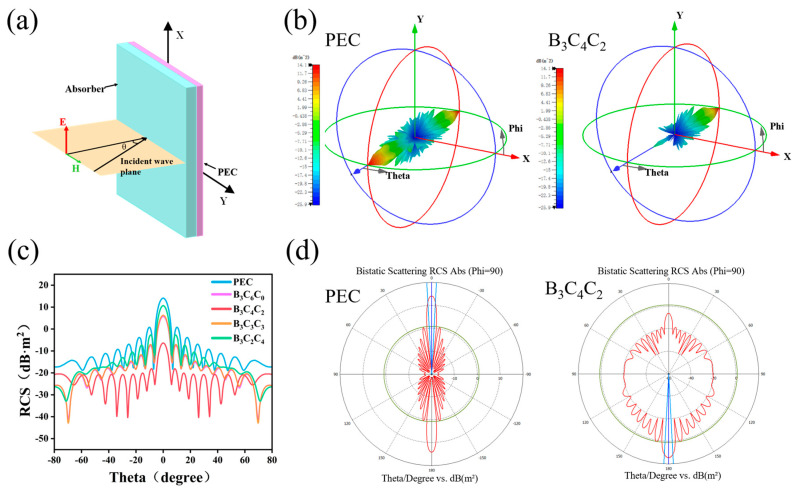
(**a**) Schematic diagram of the square simulation model; (**b**) 3D RCS distribution pattern of the reflected signal versus continuous angles; (**c**) 2D RCS comparison; (**d**) 2D RCS distribution of the reflected signal versus continuous angles.

To further evaluate the practical application of B_3_C_4_C_2_, its radar cross section (RCS) under actual far-field scattering conditions was simulated by using CST Studio Suite. A square simulation model was constructed (Figure 6a), comprising an upper aerogel attenuation layer and a bottom perfect electric conductor (PEC) substrate positioned on the XOY plane. The attenuator thickness was set to 3.6 mm, and the RCS simulation frequency was 13.5 GHz, corresponding to the optimal performance point of B_3_C_4_C_2_. The 3D and 2D RCS polar plots [44] for the PEC and B_3_C_4_C_2_ are presented in Figure 6b and Figure 6d, respectively. Generally, a lower RCS value indicates superior far-field EMW attenuation performance for EMWAs. Evidently, the PEC exhibits significant EMW reflection and scattering. In contrast, B_3_C_4_C_2_ effectively absorbs incident waves, reducing the reflected signal nearly to zero. The 2D RCS comparison (Figure 6c) clearly demonstrates that B_3_C_4_C_2_ achieves the lowest backscattering intensity within −80° to 80°, attaining a minimum RCS approaching −40 dB·m^2^. The RCS values of other samples remain around −20 dB·m^2^. These results confirm the high EMW absorption capability of B_3_C_4_C_2_ for practical radar-absorbing applications.

The EMW attenuation mechanisms of the BCC aerogel, designed specifically for high-performance absorption in flexible and wearable applications, are as follows: Targeting scenarios where minimizing secondary electromagnetic pollution is critical, the material architecture prioritizes efficient energy dissipation over reflection. This design philosophy, avoiding excessive conductive fillers that compromise impedance matching and lead to dominant reflection (as discussed in the Introduction), focuses on maximizing intrinsic loss mechanisms: (i) The long-range, continuous multi-layer micro-arch structure enhances multiple internal reflection/scattering and dissipation of electromagnetic waves. (ii) Carbonized CNC, synergizing with chitosan, establishes conductive pathways. An appropriate CNC content significantly enhances conduction loss. (iii) BNNS attached to the carbon layers induce charge accumulation at the carbon/BNNS interfaces [45]. This generates dynamic electric dipoles lagging behind the high-frequency alternating electromagnetic field, resulting in an enhanced interfacial polarization loss. (iv) The CNC introduces abundant microscopic defects and nano-interfaces within the carbon structure. These numerous defect sites further promote defect-induced polarization.

## 4. Conclusions

In summary, this work demonstrates a defect-engineering strategy via precise CNC/chitosan ratio optimization to fabricate elastic BNNS-embedded carbon aerogels. By fixing BNNS content for optimal impedance matching and modulating the CS:CNC ratio (optimized at 4:2), the aerogel simultaneously achieves a broadband microwave absorption and a robust mechanical resilience. Specifically, it shows the following: (i) an EAB of 8.3 GHz at 3.6 mm and an RL_min_ of −52.79 dB (99.999% attenuation), surpassing state-of-the-art biomass-derived EMWAs; (ii) the long-range ordered micro-arch lamellar structure leads to compressive recovery (82% height retention after 1000 cycles at 50% strain) and fatigue resistance, overcoming the fragility typical of carbon aerogels; (iii) synergistic loss mechanisms: CNC-derived topological defects and BNNS-induced interfacial polarization collaboratively enhance dielectric loss; optimal graphitization (I_D_/I_G_ = 1.08) and micro-arch lamellar structure balance conductive loss and impedance matching. The performances establish the aerogel as a premier candidate for wearable EMWAs and conformal radar-absorbing surfaces.

## Figures and Tables

**Table 1:** Composition designations of CNC/chitosan/BNNS composite suspensions with fixed BNNS content (0.3 wt%).

Sample Label	Mass Ratio (CS:CNC:BNNS)	Composition Description
B_3_C_6_C_0_	6:0:3	CS-dominated
B_3_C_4_C_2_	4:2:3	Balanced CS/CNC with BNNS
B_3_C_3_C_3_	3:3:3	Equal CS/CNC with BNNS
B_3_C_2_C_4_	2:4:3	CNC-dominated

**Table 2:** Comparison of EMW absorption performance.

Material/Sample	RL_min_ (dB)	Freq.@RL_min_ (GHz)	EAB_max_ (GHz)	Thickness@EAB_max_ (mm)	Ref./Note
This work: B_3_C_4_C_2_	−52.79	13.44	8.30	3.6	(Optimal)
This work: B_3_C_6_C_0_	−27.54	18.00	7.46	8.8	
This work: B_3_C_3_C_3_	−32.41	18.00	7.06	8.7	
This work: B_3_C_2_C_4_	−24.42	14.44	5.75	9.5	
N-doped porous carbon aerogel	−49.30	9.60	4.50	2.7	[38]
Walnut shell-derived porous carbon	−42.40	8.88	2.24	1.5	[39]
RGO/CNC/M-NP aerogels	−55.10	13.80	5.00	1.9	[40]
CF/MXene-N2	−45.00	7.50	5.00	4.5	[41]
NiFe/CoFe@C composites	−43.60	13.76	7.16	2.7	[42]
Fe_3_O_4_@Ti_3_C_2_T_x_/CNT composite	−40.1	11.40	5.8	2.0	[43]
